# Targeting Hypoxia Inducible Factors-1α As a Novel Therapy in Fibrosis

**DOI:** 10.3389/fphar.2017.00326

**Published:** 2017-05-30

**Authors:** Anji Xiong, Yi Liu

**Affiliations:** Department of Rheumatology and Immunology, West China Hospital, Sichuan UniversityChengdu, China

**Keywords:** fibrosis, chronic hypoxia, hypoxia inducible factor, targeted therapy, systemic sclerosis

## Abstract

Fibrosis, characterized by increased extracellular matrix (ECM) deposition, and widespread vasculopathy, has the prominent trait of chronic hypoxia. Hypoxia inducible factors-1α (HIF-1α), a key transcriptional factor in response to this chronic hypoxia, is involved in fibrotic disease, such as Systemic sclerosis (SSc). The implicated function of HIF-1α in fibrosis include stimulation of excessive ECM, vascular remodeling, and futile angiogenesis with further exacerbation of chronic hypoxia and deteriorate pathofibrogenesis. This review will focus on the molecular biological behavior of HIF-1α in regulating progressive fibrosis. Better understanding of the role for HIF-1α-regulated pathways in fibrotic disease will accelerate development of novel therapeutic strategies that target HIF-1α. Such new therapeutic strategies may be particularly effective for treatment of the prototypic, multisystem fibrotic, autoimmune disease SSc.

## Introduction

Fibrotic disease is a kind of chronic hypoxia related disease with pathogenesis that includes increased extracellular matrix (ECM) deposition, and widespread vasculopathy (Gabrielli et al., 2009). Fibrosis is increasingly seen as the result of deregulated tissue repair in response to chronic hypoxia that results in the excessive accumulation of ECM. Severe chronic hypoxia is overt in involved tissues of fibrotic disease patients (Distler et al., 2004). There are a variety of mechanisms leading to persistent chronic hypoxia. First, continuous and extensive microangiopathy caused by inflammation (Kahaleh, 2004; Cheung et al., 2017) or metabolic stress (Petersen et al., 2017; Wang et al., 2017) is regarded as an early and possibly the earliest pathogenic event in the fibrotic disease (Kahaleh et al., 1979) that leads to chronic hypoxia. Chronic hypoxia in turn induces vascular remodeling ultimately giving rise to progressive luminal narrowing and blockage (Flavahan et al., 2003) resulting in progressive exacerbation of the chronic hypoxic state. Moreover, excessive deposition of ECM, the hallmark of fibrosis (Bhattacharyya et al., 2012), further worsens hypoxia by increasing diffusion distances between blood vessels and tissue cells and increased tissue pressure. Extensive microangiopathy, vascular remodeling, and ECM deposition leads to vascular rarefaction and chronic hypoxia that directly contributes to progressive amplification of fibrosis. Increasing evidence has demonstrated that chronic hypoxia is actively involved in the pathogenesis of fibrosis (Ho et al., 2014) by stimulating the production of ECM including fibronectin-1, IGF-binding protein 3 (Distler et al., 2007), collagens, and collagen-modifying enzymes such as COL4A1, COL4A2, COL5A1, COL9A1, COL18A1, procollagen prolyl hydroxylases (P4HA1 and P4HA2), and lysyl hydroxylases (procollagen lysyl hydroxylase and procollagen lysyl hydroxylase 2)(Manalo et al., 2005). Hence, persistent and extensive chronic hypoxia is a distinctive feature of fibrotic disease that definitely aggravates tissue fibrosis.

Hypoxia inducible factors (HIFs) are regarded as the “master regulators” (Imtiyaz and Simon, 2010) in response to the hypoxic environment and are essential for mediating adaptive reactions to hypoxia (Appelhoff et al., 2004; Farahani et al., 2012). HIFs are in a family of basic–helix-loop-helix/Per-ARNT-Sim (bHLH/PAS) DNA binding transcription factors (Greer et al., 2012) and are heterodimers composed of two different subunits: HIF-α, that is oxygen regulated, and HIF-β, that is expressed constitutively in the nucleus (Wang et al., 1995; Semenza, 2003). There are at least three α subunits-HIF-1α, HIF-2α, and HIF-3α, that accumulate in the cytoplasm and translocate into the nucleus to form heterodimers with a β subunit. After translocating to the nucleus, the HIF heterodimers associate with co-activators and bind to hypoxia response elements (HREs) in gene promoters to initiate gene transcription (Kaelin and Ratcliffe, 2008; Semenza, 2009). Hypoxia induces stabilization and nuclear translocation of HIF-α subunits and their transcriptional activity (Kaelin and Ratcliffe, 2008) by inhibiting the activity of both prolyl hydroxylases and factor-inhibiting HIF1. Hypoxia increases the half-life of HIF-1α from 5 min to approximately 60 min (Huang et al., 1998).

Compelling evidence indicates that HIF-1α plays a key role in vascular remodeling under hypoxic conditions (Yu et al., 1999). The extensive and cumulative vascular remodeling in arterioles that accompanies chronic hypoxia results in multiple internal organ fibrosis and pulmonary hypertension (PH). Of note, PH associated with pulmonary fibrosis is the major cause of mortality in individuals suffering from fibrotic disease, such as SSc, and accumulating evidence has revealed that HIF-1α is implicated in producing excessive ECM which was the underlying cause of fibrosis (Distler et al., 2007; Higgins et al., 2007; Halberg et al., 2009; Ueno et al., 2009; Zhou et al., 2009; Gilkes et al., 2013; Nayak et al., 2016). Fibrosis is typically characterized by prolonged and/or exaggerated activation of fibroblasts (Ho et al., 2014). Strong and stable expression of HIF-1α was found in fibrotic dermal fibroblasts cultured under hypoxic conditions, 1% oxygen, equivalent to a PO_2_ value of 7 mmHg, which is close to the 10th percentile measured in involved dermal areas of fibrotic disease patients (Hong et al., 2006; Distler et al., 2007). Furthermore, increased expression of HIF-1α occurred in subcutaneous fibroblasts from healthy skin (Modarressi et al., 2010) and fibrotic skin (Hattori et al., 2015) exposed to hypoxic conditions *in vitro*. Fibroblasts isolated from human arteries also exhibited a remarkable up-regulation of HIF-1α under hypoxic conditions (Krick et al., 2005). In a more detailed study, HIF-1α completely translocated from the cytosol into the nucleus (Wenger, 2002) in dermal fibroblasts from fibrotic disease patients after hypoxic exposure (Distler et al., 2004). HIF-1α expression is elevated in a number of fibrotic diseases (Fine et al., 1998; Baan et al., 2003; Zhang et al., 2003, 2004) and overt up-regulation of HIF-1α in the skin of naïve SSc patients was observed compared with normal skin (Ioannou et al., 2013) further suggesting that HIF1α is involved in the pathogenesis of fibrotic disease, particularly in SSc (Wipff et al., 2009). In addition, HIF-1α is particularly related to subgroups of SSc patients with prominent vascular manifestations (Wipff et al., 2009). Inhibition of HIF-1α is therefore a rational strategy for novel therapeutic development since effective therapies are not yet available for fibrotic disease, such as SSc.

## Hif-1α and ECM (**Figure 1A**)

**FIGURE 1:**
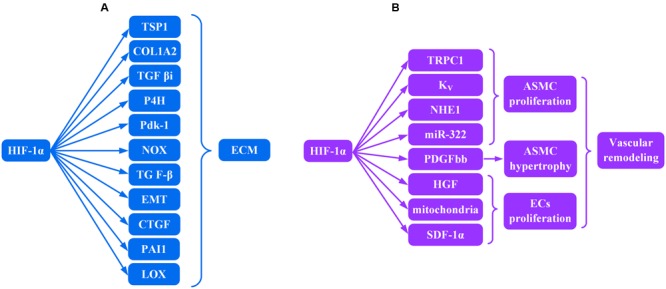
Role of HIF-lα transcription factor in the production of **(A)** ECM and **(B)** vascular remodeling.

Fibrosis is characterized by excessive deposition of ECM in organs or tissues including different kinds of collagens, hyaluronic acid, fibronectin, and proteoglycans (Ho et al., 2014). HIF-1α contributed to the up-regulated gene expression for several ECM and non-ECM in fibroblast cultures *in vitro*.

Increased expression of pro α2 (I) collagen (COL1A2), thrombospondin (TSP) 1, and transforming growth factor β–induced protein (TGF βi) were observed in both mouse embryonic and human dermal fibroblasts under hypoxic conditions (Distler et al., 2007). Bentovim et al. (2012) demonstrated that HIF-1α induced collagen hydroxylation and normal collagen secretion in the hypoxic milieu by directly activating transcription of the collagen prolyl 4-hydroxylase enzyme (P4H) and pyruvate dehydrogenase kinase 1 (Pdk1). HIF-1α deficiency resulted in impaired collagen secretion in the presence of hypoxia. Similarly, HIF-1α mediates ECM accumulation through NADPH oxidase (NOX) *in vitro* in cultured renal mesangial cells (Nayak et al., 2016). Microarray genome expression profiling from skin biopsies of fibrotic disease patients revealed that a prominent alteration in gene expression underlying fibrosis is within the transforming growth factor β (TGF-β) pathway (Whitfield et al., 2003), and TGF-β was closely involved in the induction of ECM (Falanga et al., 1987). However, HIF-1α is upstream of TGF-β production, and hypoxia-induced TGF-β production requires HIF-1α (Zhou et al., 2009). Qian et al. (2015) demonstrated that inhibition of HIF-1α reduced TGF-β expression *in vivo* as well.

Epithelial-to-mesenchymal transition (EMT) can be characterized by acquisition of mesenchymal markers such as α-smooth muscle actin (α-SMA). EMT results in the production of more ECM including α-SMA and vimentin (Strutz et al., 1995; Zeisberg and Kalluri, 2004) and requires HIF-1α expression (Zhou et al., 2009). Higgins et al. (2007) demonstrated that increased HIF-1α expression may promote fibrogenesis by facilitating EMT. Plasminogen activator inhibitor-1 (PAI-1), found in the ECM (Podor and Loskutoff, 1992) and a key inhibitor of fibrinolysis (Collen and Lijnen, 1991), inhibited proteolytic processes that were linked with fibrosis (Eddy et al., 1995). HIF-1α heterodimers with HIF-1β induced by hypoxia *in vivo* bind HRE in the PAI-1 promoter and induce PAI-1 expression (Kietzmann et al., 1999). Lysyl oxidase (LOX) is important for normal synthesis of collagen and elastin (Giampuzzi et al., 2000; Oleggini et al., 2007). LOX is a transcriptional target for HIF-1α-HIF-1β heterodimers (Halberg et al., 2009) that translocate into the nuclear compartment of fibrogenic cells (Li et al., 1997) and is up-regulated during fibrogenesis. Higgins et al. (2007), Halberg et al. (2009) showed that HIF-1α could up-regulate the expression of LOX *in vivo* and *in vitro*, leading to the accumulation of collagen and other components involved in establishing and remodeling the ECM. Halberg et al. (2009) pinpointed LOX as a key player in HIF-1α mediated deposition of ECM. Furthermore, connective tissue growth factor (CTGF) has been reported to enhance cell proliferation and ECM production in fibroblasts (Frazier et al., 1996). Mounting evidence has demonstrated that expression of CTGF is upregulated during fibrotic disorders (Igarashi et al., 1996; Ito et al., 1998; Leask et al., 2002; Higgins et al., 2003), and in hypoxia, the induction of CTGF is directly mediated by HIF-1α-HIF-1β heterodimer binding to the CTGF associated HRE (Higgins et al., 2004). In summary, HIF-1α is ubiquitous in many different tissues (Wang et al., 1995) and in fibrotic disease contributes to persistent pathofibrogenesis in multiple organs by stimulating production of excessive ECM.

## HIF-1α and Vascular Remodeling (**Figure 1B**)

Vascular remodeling is primarily composed of dysregulated proliferation of endothelial cells (ECs) and an increase in the number (hyperplasia) and volume (hypertrophy) of arterial smooth muscle cells (ASMC) resulting in progressive vascular occlusion and chronic hypoxia. High expression of HIF-1α within endothelial plexiform lesions (Tuder et al., 2001) and ASMC (Bonnet et al., 2006) suggests a strong correlation between HIF-1α and proliferative vasculopathy.

Arterial smooth muscle cells hyperproliferation in the media of the artery was suggested to be the key event in vascular remodeling (Cheng et al., 2017). Transient receptor potential channel (TRPC) 1, a non-selective cation channel, is permeable to Ca^2+^ ions. Increase in levels of TRPC1 mediated by bone morphogenetic protein4 (BMP4) (Wang et al., 2015) was HIF-1α dependent in ASMC (Wang et al., 2006). Reduction in voltage-gated K^+^ currents, resulting in membrane depolarization and activation of voltage-dependent Ca^2+^ channels and subsequently increasing Ca^2+^ influx, was regulated by HIF-1α as well (Shimoda et al., 2001). Both voltage-gated K^+^ (Kv) channels and TRPC1, mediated by HIF-1α, contributed to an increase in cytosolic free Ca^2+^ which was a major trigger for ASMC proliferation (Veith et al., 2016). ASMC proliferation may be a consequence of up-regulated aquaporin 1 as a result of the increased cytosolic free Ca^2+^ (Yun et al., 2015). Furthermore, both TRPC1 silencing by small interfering RNA (siRNA) and TRPC1 knockout impaired hypoxia-induced ASMC proliferation *in vitro*, and TRPC1^-/-^ mice had less vascular muscularization compared with wild type mice (Malczyk et al., 2013). In addition, hypoxic induction of the Na^+^/H^+^ exchanger isoform 1 (NHE1) expression and alkalinization of intracellular pH were regulated by HIF-1α (Shimoda et al., 2006). Both activation of the Na^+^/H^+^ exchanger and alkalinization of intracellular pH were necessary for ASMC proliferation (Quinn et al., 1996). Zeng et al. (2015) demonstrated that HIF-1α transcriptionally upregulated the expression of miR-322 in hypoxia, which led to proliferative responses of ASMC due to direct targeting of BMPR1a and smad5. Similarly, Platelet derived growth factor bb (PDGFbb) can induce proliferation of ASMC *in vitro* and *in vivo* (Schermuly et al., 2005). PDGFbb-induced signaling gave rise to the hypertrophy of ASMC both *in vitro* and *in vivo* (Ke et al., 2016) via excessive deposition of hyaluronic acid (HA) in smooth muscle cells (Pullen et al., 2001). The possible mechanism is through tyrosine 31 (Y31) and 118 (Y118) phosphorylation of paxillin, which was attenuated by HIF-1α knockdown (Veith et al., 2014).

Similarly, HIF-1α is also involved in the proliferation of ECs. Abnormally proliferating ECs are characterized by low numbers of mitochondria (Xu et al., 2007). Knockdown of HIF-1α increased the numbers of mitochondria in ECs *in vitro* (Fijalkowska et al., 2010) and suggests that the reduced mitochondria number in abnormally proliferating ECs may be a consequence, at least in part, of increased HIF-1α expression. HIF -1α inducible factors include hepatocyte growth factor (HGF) (Kitajima et al., 2008) and stromal-derived factor-1a (SDF-1a) (Ceradini et al., 2004). A special kind of hematopoietic endothelial stem cell, CD34^+^CD133^+^hemangioblast, may promote angioproliferative vascular remodeling (Asosingh et al., 2008). Local production of chemoattractants, such as SDF-1α and HGF, by diseased endothelium can recruit substantial numbers of CD34^+^CD133^+^hemangioblasts to sites of angioproliferative vascular remodeling (Farha et al., 2011). Both signal transducers and activators of transcription (STAT) 3 (Xu and Erzurum, 2011) and chloride intracellular channel 4 (CLIC4) (Wojciak-Stothard et al., 2014) contribute to the hyperproliferative pathology of ECs invoking another important role for HIF-1α in vascular fibrosis.

## Targeting HIF-1α in Fibrosis

Studies to date indicate that HIF-1α is intimately involved in persistent pathofibrogenesis, vascular remodeling, and PH in fibrotic disease. Severe, multiple organ fibrosis associated with the continuous accumulation of HIF-1α, caused by chronic or prolonged hypoxia in fibrotic disease, suggests that HIF-1α maybe a promising target for novel fibrotic disease treatments, such as SSc.

Recently, hypoxic prodrugs, projecting to be specifically activated in the low O_2_ milieu, deliver the active agent to hypoxic tissues through reduction of the prodrug by cellular reductases (Phillips, 2016). These hypoxic prodrug agents may significantly alleviate off-target effects of the biological therapy by limiting active drug to hypoxic tissue and only inhibiting HIF-1α in hypoxic tissues. Gene therapy targeting HIF-1α may also be effective for therapy in hypoxia-related diseases as well (Tal et al., 2008; Wang et al., 2008; del Rey et al., 2009; Chen et al., 2016). In addition, the therapeutic benefits of HIF-1α inhibitors would be maximized in the presence of delivery carriers that eliminate pharmacokinetic and stability problems and minimize potential systemic toxicity. For example, liposomes and nanoscale-based drug delivery systems may be applied as a delivery assistant for HIF-1α gene therapy (Wang et al., 2008; Chen et al., 2016). The most successful example of a successful liposomal drug delivery system may be that for Amphotericin B, which has been widely applied in the clinic for treating invasive fungal infections. Amphotericin B is a highly effective drug but with potential severe toxic side effects (Barratt and Bretagne, 2007; Wasko et al., 2012). Amphotericin B encapsulated in liposome has significantly reduced toxicity as well as increased therapeutic benefit when administered systemically encapsulated within liposomes (Torchilin, 2005; Allen and Cullis, 2013). Antisense oligonucleotides targeted to HIF-1α mRNA combined with doxorubicin were successfully delivered to oncocytes by poly (ethylene glycol) polymer (PEGylated) liposomes as drug carriers (Wang et al., 2008). Furthermore, YC-1 [3-(5′-hydroxymethyl-2′-furyl)-1-benzyl indazole], a HIF-1α inhibitor, reduced ECM accumulation *in vivo* (Nayak et al., 2016). Trichostatin A, identified indirectly to down-regulate HIF-1α, has been applied in clinical trials in patients with cancers (Kim et al., 2001) and has been shown to reduce the release of collagen from fibrotic dermal fibroblasts *in vitro* (Huber et al., 2005). In conclusion, a viable therapy option for fibrotic disease may include agents that target and inhibit HIF-1α since delivery vehicles may help reduce off-target effects and enhance therapeutic efficiency (Sercombe et al., 2015).

On the other hand, HIF-1α has been repeatedly observed to assist wound healing through inflammation, angiogenesis, vasculargenesis, and fibroplasia in acute injury (Semenza, 1998, 1999). The most prominent contraindication for systemic administration of HIF-1α inhibitors, therefore, is trauma. An analogy for adverse effects that may accompany biological therapy to inhibit HIF-1α may be tumor necrosis factor-α (TNF-α) inhibitors in rheumatic diseases. TNF-α inhibitors inhibit inflammation that is necessary for tissue repair. HIF-1α is required for repair in acute injury as well (Darby and Hewitson, 2016). In particular, we need to be vigilant about physiological repair events such as menstruation within the context of HIF-1α inhibitor administration.

Persistent and remarkable up-regulation of vascular endothelial growth factor (VEGF) has been observed in all stages of fibrotic disease (Distler et al., 2004) and actively proliferating ECs of plexiform lesions (Tuder et al., 2001). VEGF is the predominant proangiogenic factor regulated by HIF-1α in other hypoxia related diseases, but VEGF up-regulation and consequent massive and extensive microangiopathy in fibrotic disease is HIF-1α independent, even with hypoxia (Distler et al., 2004). HIF-1α expression did not correlate with up-regulated VEGF in affected tissues from patients suffering from fibrotic disease (Distler et al., 2004). Since HIF-1α is critical for VEGF up-regulation in other hypoxia related diseases, consideration must be given to the question of whether deceased VEGF after systemic HIF-1α inhibition may severely decrease and impair neoangiogenesis. The worry may be unnecessary for fibrotic disease and its associated massive and extensive microangiopathy in affected tissues and organs. Up-regulated expression of VEGF is also driven by interleukin-1β, PDGF, and TGF-β, all of which are up-regulated in fibrotic disease and can stimulate the expression of VEGF (Pertovaara et al., 1994; Kissin and Korn, 2003). Moreover, the role played by HIF-2α and HIF-3α in the over-expression of VEGF has not yet been extensively investigated. Above all, sufficient tissue vascularization depends on strict regulation of VEGF expression rather than on persistent up-regulated expression of VEGF (Distler et al., 2004). The formation of chaotic vessels, feathered with glomeruloid and haemangioma-like morphology, was partly due to chronic and uncontrolled over-expression of VEGF (Drake and Little, 1995; Sundberg et al., 2001). Dor et al. (2002) designed an animal model system in which a source of VEGF could be specifically induced and steadily maintained for a desired duration and then subsequently switched off. Time-dependent regulation of VEGF expression was necessary for adequate and normal vascularization (Dor et al., 2002). Persistent, uninterrupted exposure to VEGF led to formation of irregularly shaped, sac-like vessels resulting in decreased blood flow compared to normal, mature, functional blood vessel formation after short-term over-expression of VEGF (Dor et al., 2002). Irregularly shaped, sac-like vessels observed in nailfold, a prominent character of the prototypic fibrotic disease**-**SSc (LeRoy, 1996), may also suggest that persistent up-regulated expression of VEGF is involved in fibrotic disease, and is harmful rather than beneficial, regardless of whether VEGF is HIF-1α independent in fibrosis (Distler et al., 2004). Other angiogenic factors contribute less to neovascularization and have no effect on irregular and sac-like vessels in the presence of persistent and remarkable up-regulation of VEGF. The US FDA approved FTY720 inhibits HIF-1α accumulation by inhibiting the S1P signaling pathway. FTY720 transformed a chaotic vascular network to vascular normalization while simultaneously and subsequently redressing hypoxia *in vivo* and *in vitro* (Gstalder et al., 2016). That result gives further credence to the suggestion that HIF-1α is implicated in chaotic angiogenesis. This result also suggests that targeting HIF-1α would be a viable strategy for fibrotic disease, such as SSc, without impairing normal angiogenesis.

## Conclusion

**FIGURE 2:**
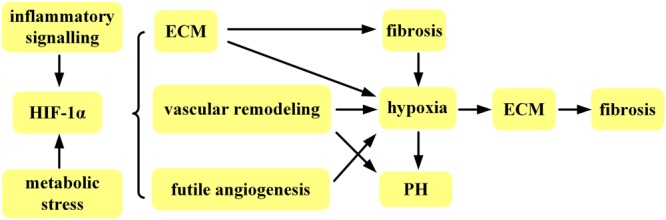
Simplified overview of HIF-1α implicated in fibrotic disease.

HIF-1α *per se* is helpful in repairing injury and correcting hypoxia via multiple mechanisms, however, prolonged exposure to HIF-1α is harmful and contributes to persistent pathofibrogenesis in fibrotic disease (**Figure 2**). Furthermore, fibrosis in organs resulting in organ failure accounts for much of the morbidity and mortality associated with fibrotic disease. SSc is prototypic multisystem fibrotic disease and present immunosuppressive therapy exhibits intolerable side effects without selectively targeting the immunopathogenic mechanisms responsible for SSc. In addition, fibrosis in SSc is not restricted to a single organ, but rather involves multiple internal organs and skin. Biotherapy targeting HIF-1α, therefore, is a promising therapeutic alternative that is more likely to confer therapeutic benefits specific to fibrotic disease, particularly to SSc, by attenuating fibrosis and terminating or delaying vascular remodeling.

## Author Contributions

AX wrote and assembled the manuscript. YL prepared the figures and revised the manuscript.

## Conflict of Interest Statement

The authors declare that the research was conducted in the absence of any commercial or financial relationships that could be construed as a potential conflict of interest.

## References

[B1] Allen T. M. Cullis P. R. (2013). Liposomal drug delivery systems: From concept to clinical applications. *Adv. Drug Deliv. Rev.* 65 36–48. 10.1016/j.addr.2012.09.037 23036225

[B2] Appelhoff R. J. Tian Y. M. Raval R. R. Turley H. Harris A. L. Pugh C. W. (2004). Differential function of the prolyl hydroxylases PHD1, PHD2, and PHD3 in the regulation of hypoxia-inducible factor. *J. Biol. Chem.* 279 38458–38465. 10.1074/jbc.M406026200 15247232

[B3] Asosingh K. Aldred M. A. Vasanji A. Drazba J. Sharp J. Farver C. (2008). Circulating angiogenic precursors in idiopathic pulmonary arterial hypertension. *Am. J. Pathol.* 172 615–627. 10.2353/ajpath.2008.070705 18258847 PMC2258264

[B4] Baan C. van Gelder T. Peeters A. Mol W. Niesters H. Weimar W. (2003). Living kidney donors and hypoxia-inducible factor-1 alpha. *Transplantation* 75 570–571. 10.1097/01.tp.0000034241.55602.ad 12605132

[B5] Barratt G. Bretagne S. (2007). Optimizing efficacy of Amphotericin B through nanomodification. *Int. J. Nanomed.* 2 301–313. PMC2676657 18019830

[B6] Bentovim L. Amarilio R. Zelzer E. (2012). HIF1 alpha is a central regulator of collagen hydroxylation and secretion under hypoxia during bone development. *Development* 139 4473–4483. 10.1242/dev.083881 23095889

[B7] Bhattacharyya S. Wei J. Varga J. (2012). Understanding fibrosis in systemic sclerosis: shifting paradigms, emerging opportunities. *Nat. Rev. Rheumatol.* 8 42–54. 10.1038/nrrheum.2011.149 PMC3954787 22025123

[B8] Bonnet S. Michelakis E. D. Porter C. J. Andrade-Navarro M. A. Thebaud B. Bonnet S. (2006). An abnormal mitochondrial-hypoxia inducible factor-1 alpha-Kv channel pathway disrupts oxygen sensing and triggers pulmonary arterial hypertension in fawn hooded rats - Similarities to human pulmonary arterial hypertension. *Circulation* 113 2630–2641. 10.1161/circulationaha.105.609008 16735674

[B9] Ceradini D. J. Kulkarni A. R. Callaghan M. J. Tepper O. M. Bastidas N. Kleinman M. E. (2004). Progenitor cell trafficking is regulated by hypoxic gradients through HIF-1 induction of SDF-1. *Nat. Med.* 10 858–864. 10.1038/nm1075 15235597

[B10] Chen Z. Zhang T. Wu B. Zhang X. (2016). Insights into the therapeutic potential of hypoxia-inducible factor-1 alpha small interfering RNA in malignant melanoma delivered via folate-decorated cationic liposomes. *Int. J. Nanomed.* 11 991–1002. 10.2147/ijn.s101872 PMC4795592 27042054

[B11] Cheng G. Wang X. Li Y. He L. (2017). Let-7a-transfected mesenchymal stem cells ameliorate monocrotaline-induced pulmonary hypertension by suppressing pulmonary artery smooth muscle cell growth through STAT3-BMPR2 signaling. *Stem cell Res. Ther.* 8:34 10.1186/s13287-017-0480-y PMC5303212 28187784

[B12] Cheung A. C. Lazaridis K. N. LaRusso N. F. Gores G. J. (2017). Emerging pharmacologic therapies for primary sclerosing cholangitis. *Curr. Opin. Gastroenterol.* 33 149–157. 10.1097/mog.0000000000000352 28257308 PMC5646688

[B13] Collen D. Lijnen H. R. (1991). Basic and clinical aspects of fibrinolysis and thrombolysis. *Blood* 78 3114–3124. 1742478

[B14] Darby I. A. Hewitson T. D. (2016). Hypoxia in tissue repair and fibrosis. *Cell Tissue Res.* 365 553–562. 10.1007/s00441-016-2461-3 27423661

[B15] del Rey M. J. Izquierdo E. Caja S. Usategui A. Santiago B. Galindo M. (2009). Human inflammatory synovial fibroblasts induce enhanced myeloid cell recruitment and angiogenesis through a hypoxia-inducible transcription factor 1 alpha/vascular endothelial growth factor-mediated pathway in immunodeficient mice. *Arthritis Rheumatol.* 60 2926–2934. 10.1002/art.24844 19790065

[B16] Distler J. H. W. Juengel A. Pileckyte M. Zwerina J. Michel B. A. Gay R. E. (2007). Hypoxia-induced increase in the production of extracellular matrix proteins in systemic sclerosis. *Arthritis Rheum.* 56 4203–4215. 10.1002/art.23074 18050252

[B17] Distler O. Distler J. H. W. Scheid A. Acker T. Hirth A. Rethage J. (2004). Uncontrolled expression of vascular endothelial growth factor and its receptors leads to insufficient skin angiogenesis in patients with systemic sclerosis. *Circ. Res.* 95 109–116. 10.1161/01.res.0000134644.89917.96 15178641

[B18] Dor Y. Djonov V. Abramovitch R. Itin A. Fishman G. I. Carmeliet P. (2002). Conditional switching of VEGF provides new insights into adult neovascularization and pro-angiogenic therapy. *Embo J.* 21 1939–1947. 10.1093/emboj/21.8.1939 11953313 PMC125962

[B19] Drake C. J. Little C. D. (1995). Exogenous vascular endothelial growth-factor induces malformed and hyperfused vessels during embryonic neovascularization. *Proc. Natl. Acad. Sci. U.S.A.* 92 7657–7661. 10.1073/pnas.92.17.7657 7543999 PMC41204

[B20] Eddy A. A. Giachelli C. M. McCulloch L. Liu E. (1995). Renal expression of genes that promote interstitial inflammation and fibrosis in rats with protein-overload proteinuria. *Kidney Int.* 47 1546–1557. 10.1038/ki.1995.218 7643523

[B21] Falanga V. Tiegs S. L. Alstadt S. P. Roberts A. B. Sporn M. B. (1987). Transforming growth-factor-beta - selective increase in glycosaminoglycan synthesis by cultures of fibroblasts from patients with progressive systemic-sclerosis. *J. Invest. Dermatol.* 89 100–104. 10.1111/1523-1747.ep12580445 3496398

[B22] Farahani R. M. Sarrafpour B. Simonian M. Li Q. Hunter N. (2012). Directed glia-assisted angiogenesis in a mature neurosensory structure: pericytes mediate an adaptive response in human dental pulp that maintains blood-barrier function. *J. Compar. Neurol.* 520 3803–3826. 10.1002/cne.23162 22678627

[B23] Farha S. Asosingh K. Xu W. Sharp J. George D. Comhair S. (2011). Hypoxia-inducible factors in human pulmonary arterial hypertension: a link to the intrinsic myeloid abnormalities. *Blood* 117 3485–3493. 10.1182/blood-2010-09-306357 21258008 PMC3072874

[B24] Fijalkowska I. Xu W. Comhair S. A. A. Janocha A. J. Mavrakis L. A. Krishnamachary B. (2010). Hypoxia inducible-factor1 alpha regulates the metabolic shift of pulmonary hypertensive endothelial cells. *Am. J. Pathol.* 176 1130–1138. 10.2353/ajpath.2010.090832 20110409 PMC2832136

[B25] Fine L. G. Orphanides C. Norman J. T. (1998). Progressive renal disease: the chronic hypoxia hypothesis. *Kidney Int.* 65 S74–S78. 9551436

[B26] Flavahan N. A. Flavahan S. Mitra S. Chotani M. A. (2003). The vasculopathy of Raynaud’s phenomenon and scleroderma. *Rheum. Dis. Clin. North Am.* 29 275–91 vi 10.1016/s0889-857x(03)00021-8 12841295

[B27] Frazier K. Williams S. Kothapalli D. Klapper H. Grotendorst G. R. (1996). Stimulation of fibroblast cell growth, matrix production, and granulation tissue formation by connective tissue growth factor. *J. Invest. Dermatol.* 107 404–411. 10.1111/1523-1747.ep12363389 8751978

[B28] Gabrielli A. Avvedimento E. V. Krieg T. (2009). Mechanisms of disease: scleroderma. *N. Engl. J. Med.* 360 1989–2003. 10.1056/NEJMra0806188 19420368

[B29] Giampuzzi M. Botti G. Di Duca M. Arata L. Ghiggeri G. Gusmano R. (2000). Lysyl oxidase activates the transcription activity of human collagene III promoter - Possible involvement of Ku antigen. *J. Biol. Chem.* 275 36341–36349. 10.1074/jbc.M003362200 10942761

[B30] Gilkes D. M. Bajpai S. Chaturvedi P. Wirtz D. Semenza G. L. (2013). Hypoxia-inducible factor 1 (HIF-1) promotes extracellular matrix remodeling under hypoxic conditions by inducing P4HA1, P4HA2, and PLOD2 expression in fibroblasts. *J. Biol. Chem.* 288 10819–10829. 10.1074/jbc.M112.442939 23423382 PMC3624462

[B31] Greer S. N. Metcalf J. L. Wang Y. Ohh M. (2012). The updated biology of hypoxia-inducible factor. *Embo J.* 31 2448–2460. 10.1038/emboj.2012.125 22562152 PMC3365421

[B32] Gstalder C. Ader I. Cuvillier O. (2016). FTY720 (Fingolimod) inhibits HIF1 and HIF2 signaling, promotes vascular remodeling, and chemosensitizes in renal cell carcinoma animal model. *Mol. Cancer Ther.* 15 2465–2474. 10.1158/1535-7163.mct-16-0167 27507852

[B33] Halberg N. Khan T. Trujillo M. E. Wernstedt-Asterholm I. Attie A. D. Sherwani S. (2009). Hypoxia-inducible factor 1 alpha induces fibrosis and insulin resistance in white adipose tissue. *Mol. Cell. Biol.* 29 4467–4483. 10.1128/mcb.00192-09 19546236 PMC2725728

[B34] Hattori M. Yokoyama Y. Hattori T. Motegi S.-I. Amano H. Hatada I. (2015). Global DNA hypomethylation and hypoxia-induced expression of the ten eleven translocation (TET) family, TET1, in scleroderma fibroblasts. *Exp. Dermatol.* 24 841–846. 10.1111/exd.12767 26013976

[B35] Higgins D. F. Biju M. P. Akai Y. Wutz A. Johnson R. S. Haase V. H. (2004). Hypoxic induction of Ctgf is directly mediated by Hif-1. *Am. J. Physiol. Renal Physiol.* 287 F1223–F1232. 10.1152/ajprenal.00245.2004 15315937

[B36] Higgins D. F. Kimura K. Bernhardt W. M. Shrimanker N. Akai Y. Hohenstein B. (2007). Hypoxia promotes fibrogenesis in vivo via HIF-1 stimulation of epithelial-to-mesenchymal transition. *J. Clin. Invest.* 117 3810–3820. 10.1172/jci30487 18037992 PMC2082142

[B37] Higgins D. F. Lappin D. W. P. Kieran N. E. Anders H. J. Watson R. W. G. Strutz F. (2003). DNA oligonucleotide microarray technology identifies fisp-12 among other potential fibrogenic genes following murine unilateral ureteral obstruction (UUO): modulation during epithelial-mesenchymal transition. *Kidney Int.* 64 2079–2091. 10.1046/j.1523-1755.2003.00306.x 14633130

[B38] Ho Y. Y. Lagares D. Tager A. M. Kapoor M. (2014). Fibrosis-a lethal component of systemic sclerosis. *Nat. Rev. Rheumatol.* 10 390–402. 10.1038/nrrheum.2014.53 24752182

[B39] Hong K. H. Yoo S. A. Kang S. S. Choi J. J. Kim W. U. Cho C. S. (2006). Hypoxia induces expression of connective tissue growth factor in scleroderma skin fibroblasts. *Clin. Exp. Immunol.* 146 362–370. 10.1111/j.1365-2249.2006.03199.x 17034590 PMC1942060

[B40] Huang L. E. Gu J. Schau M. Bunn H. F. (1998). Regulation of hypoxia-inducible factor 1 alpha is mediated by an O-2-dependent degradation domain via the ubiquitin-proteasome pathway. *Proc. Natl. Acad. Sci. U.S.A.* 95 7987–7992. 10.1073/pnas.95.14.7987 9653127 PMC20916

[B41] Huber L. C. Distler J. H. W. Michel B. A. Gay R. E. Kalden J. R. Matucci-Cerinic M. (2005). Inhibition of histone deacetylases reduces the TGF beta-stimulated production of extracellular matrix proteins of skin fibroblasts from patients with systemic sclerosis (SSC). *Arthritis Rheum.* 52 S463–S463.

[B42] Igarashi A. Nashiro K. Kikuchi K. Sato S. Ihn H. Fujimoto M. (1996). Connective tissue growth factor gene expression in tissue sections from localized scleroderma, keloid, and other fibrotic skin disorders. *J. Invest. Dermatol.* 106 729–733. 10.1111/1523-1747.ep12345771 8618012

[B43] Imtiyaz H. Z. Simon M. C. (2010). “Hypoxia-inducible factors as essential regulators of inflammation,” in *Diverse Effects of Hypoxia on Tumor Progression* ed. Simon M. C. (Berlin: Springer). 10.1007/82_2010_74 PMC3144567 20517715

[B44] Ioannou M. Pyrpasopoulou A. Simos G. Paraskeva E. Nikolaidou C. Venizelos I. (2013). Upregulation of VEGF expression is associated with accumulation of HIF-1 alpha in the skin of naive scleroderma patients. *Mod. Rheumatol.* 23 1245–1248. 10.1007/s10165-012-0787-6 23096096

[B45] Ito Y. Aten J. Bende R. J. Oemar B. S. Rabelink T. J. Weening J. J. (1998). Expression of connective tissue growth factor in human renal fibrosis. *Kidney Int.* 53 853–861. 10.1046/j.1523-1755.1998.00820.x 9551391

[B46] Kaelin W. G. Jr. Ratcliffe P. J. (2008). Oxygen sensing by metazoans: the central role of the HIF hydroxylase pathway. *Mol. Cell.* 30 393–402. 10.1016/j.molcel.2008.04.009 18498744

[B47] Kahaleh M. B. (2004). Raynaud phenomenon and the vascular disease in scleroderma. *Curr. Opin. Rheumatol.* 16 718–722. 10.1097/01.bor.0000138677.88694.a4 15577610

[B48] Kahaleh M. B. Sherer G. K. Leroy E. C. (1979). Endothelial injury in scleroderma. *J. Exp. Med.* 149 1326–1335. 10.1084/jem.149.6.1326 312896 PMC2184886

[B49] Ke M.-W. Cheng C.-C. Huang W.-C. Wann S.-R. Shu C.-W. Tseng C.-J. (2016). Suppression of HIF-1 alpha-mediated PDGF(BB) signaling inhibits the hypertrophy of pulmonary arterial smooth muscle cell in vitro and in vivo. *Circ. Res.* 119 E170–E170.

[B50] Kietzmann T. Roth U. Jungermann K. (1999). Induction of the plasminogen activator inhibitor-1 gene expression by mild hypoxia via a hypoxia response element binding the hypoxia-inducible factor-1 in rat hepatocytes. *Blood* 94 4177–4185. 10590062

[B51] Kim M. S. Kwon H. J. Lee Y. M. Baek J. H. Jang J. E. Lee S. W. (2001). Histone deacetylases induce angiogenesis by negative regulation of tumor suppressor genes. *Nat. Med.* 7 437–443. 10.1038/86507 11283670

[B52] Kissin E. Y. Korn J. H. (2003). Fibrosis in scleroderma. *Rheum. Dis. Clin. North Am.* 29 351–369. 10.1016/s0889-857x(03)00018-8 12841299

[B53] Kitajima Y. Ide T. Ohtsuka T. Miyazaki K. (2008). Induction of hepatocyte growth factor activator gene expression under hypoxia activates the hepatocyte growth factor/c-Met system via hypoxia inducible factor-1 in pancreatic cancer. *Cancer Sci.* 99 1341–1347. 10.1111/j.1349-7006.2008.00828.x 18422749 PMC11159873

[B54] Krick S. Hanze J. Eul B. Savai R. Seay U. Grimminger F. (2005). Hypoxia-driven proliferation of human pulmonary artery fibroblasts: cross-talk between HIF-1 alpha and an autocrine angiotensin system. *FASEB J.* 19 857–859. 10.1096/fj.04-2890fje 15718424

[B55] Leask A. Holmes A. Abraham D. J. (2002). Connective tissue growth factor: a new and important player in the pathogenesis of fibrosis. *Curr. Rheumatol. Rep.* 4 136–142. 10.1007/s11926-002-0009-x 11890879

[B56] LeRoy E. C. (1996). Systemic sclerosis - A vascular perspective. *Rheum. Dis. Clin. North Am.* 22 675–694. 10.1016/S0889-857X(05)70295-7 8923590

[B57] Li W. D. Nellaiappan K. Strassmaier T. Graham L. Thomas K. M. Kagan H. M. (1997). Localization and activity of lysyl oxidase within nuclei of fibrogenic cells. *Proc. Natl. Acad. Sci. U.S.A.* 94 12817–12822. 10.1073/pnas.94.24.12817 9371758 PMC24221

[B58] Malczyk M. Veith C. Fuchs B. Hofmann K. Storch U. Schermuly R. T. (2013). Classical transient receptor potential channel 1 in hypoxia-induced pulmonary hypertension. *Am. J. Respir. Crit. Care Med.* 188 1451–1459. 10.1164/rccm.201307-1252OC 24251695

[B59] Manalo D. J. Rowan A. Lavoie T. Natarajan L. Kelly B. D. Ye S. Q. (2005). Transcriptional regulation of vascular endothelial cell responses to hypoxia by HIF-1. *Blood* 105 659–669. 10.1182/blood-2004-07-2958 15374877

[B60] Modarressi A. Pietramaggiori G. Godbout C. Vigato E. Pittet B. Hinz B. (2010). Hypoxia impairs skin myofibroblast differentiation and function. *J. Investig. Dermatol.* 130 2818–2827. 10.1038/jid.2010.224 20686497

[B61] Nayak B. K. Shanmugasundaram K. Friedrichs W. E. Cavaglierii R. C. Patel M. Barnes J. (2016). HIF-1 mediates renal fibrosis in OVE26 type 1 diabetic mice. *Diabetes Metab. Res. Rev.* 65 1387–1397. 10.2337/db15-0519 PMC4839204 26908870

[B62] Oleggini R. Gastaldo N. Di Donato A. (2007). Regulation of elastin promoter by lysyl oxidase and growth factors: cross control of lysyl oxidase on TGF-beta 1 effects. *Matrix Biol.* 26 494–505. 10.1016/j.matbio.2007.02.003 17395448

[B63] Pertovaara L. Kaipainen A. Mustonen T. Orpana A. Ferrara N. Saksela O. (1994). Vascular endothelial growth-factor is induced in response to transforming growth-factor-beta in fibroblastic and epithelial-cells. *J. Biol. Chem.* 269 6271–6274. 8119973

[B64] Petersen P. S. Lei X. Wolf R. M. Rodriguez S. Tan S. Y. Little H. C. (2017). CTRP7 deletion attenuates obesity-linked glucose intolerance, adipose tissue inflammation, and hepatic stress. *Am. J. Physiol. Endocrinol. Metab.* 312 E309–E325. 10.1152/ajpendo.00344.2016 28223291 PMC5406989

[B65] Phillips R. M. (2016). Targeting the hypoxic fraction of tumours using hypoxia-activated prodrugs. *Cancer Chemother. Pharmacol.* 77 441–457. 10.1007/s00280-015-2920-7 26811177 PMC4767869

[B66] Podor T. J. Loskutoff D. J. (1992). Immunoelectron microscopic localization of type 1 plasminogen activator inhibitor in the extracellular matrix of transforming growth factor-β-activated endothelial cells. *Ann. N.Y. Acad. Sci.* 667 46–49. 10.1111/j.1749-6632.1992.tb51595.x 1309070

[B67] Pullen M. A. Thomas K. Wu H. L. Nambi P. (2001). Stimulation of hyaluronan synthetase by platelet-derived growth factor bb in human prostate smooth muscle cells. *Pharmacology* 62 103–106. 10.1159/000056079 11174080

[B68] Qian F. He M. Duan W. Mao L. Li Q. Yu Z. (2015). Cross regulation between hypoxia-inducible transcription factor-1 alpha (HIF-1 alpha) and transforming growth factor (TGF)-beta 1 mediates nickel oxide nanoparticles (NiONPs)-induced pulmonary fibrosis. *Am. J. Trans. Res.* 7 2364–2378. PMC4697716 26807184

[B69] Quinn D. A. Dahlberg C. G. W. Bonventre J. P. Scheid C. R. Honeyman T. Joseph P. M. (1996). The role of Na+/H+ exchange and growth factors in pulmonary artery smooth muscle cell proliferation. *Am. J. Respir. Cell Mol. Biol.* 14 139–145. 10.1165/ajrcmb.14.2.8630263 8630263

[B70] Schermuly R. T. Dony E. Ghofrani H. A. Pullamsetti S. Savai R. Roth M. (2005). Reversal of experimental pulmonary hypertension by PDGF inhibition. *J. Clin. Investig.* 115 2811–2821. 10.1172/jci24838 16200212 PMC1236676

[B71] Semenza G. L. (1998). Hypoxia-inducible factor 1: master regulator of O-2 homeostasis. *Curr. Opin. Genet. Dev.* 8 588–594. 10.1016/S0959-437X(98)80016-6 9794818

[B72] Semenza G. L. (1999). Regulation of mammalian O-2 homeostasis by hypoxia- inducible factor 1. *Annu. Rev. Cell Dev. Biol.* 15 551–578. 10.1146/annurev.cellbio.15.1.551 10611972

[B73] Semenza G. L. (2003). Targeting HIF-1 for cancer therapy. *Nat. Rev. Cancer* 3 721–732. 10.1038/nrc1187 13130303

[B74] Semenza G. L. (2009). Regulation of vascularization by hypoxia-inducible factor 1. *Ann. N.Y. Acad. Sci.* 1177 2–8. 10.1111/j.1749-6632.2009.05032.x 19845601

[B75] Sercombe L. Veerati T. Moheimani F. Wu S. Y. Sood A. K. Hua S. (2015). Advances and challenges of liposome assisted drug delivery. *Front. Pharmacol.* 6:286 10.3389/fphar.2015.00286 PMC4664963 26648870

[B76] Shimoda L. A. Fallon M. Pisarcik S. Wang J. Semenza G. L. (2006). HIF-1 regulates hypoxic induction of NHE1 expression and alkalinization of intracellular pH in pulmonary arterial myocytes. *Am. J. Physiol. Lung Cell. Mol. Physiol.* 291 L941–L949. 10.1152/ajplung.00528.2005 16766575

[B77] Shimoda L. A. Manalo D. J. Sham J. S. K. Semenza G. L. Sylvester J. T. (2001). Partial HIF-1 alpha deficiency impairs pulmonary arterial myocyte electrophysiological responses to hypoxia. *Am. J. Physiol. Lung Cell. Mol. Physiol.* 281 L202–L208. 11404263 10.1152/ajplung.2001.281.1.L202

[B78] Strutz F. Okada H. Lo C. W. Danoff T. Carone R. L. Tomaszewski J. E. (1995). Identification and characterization of a fibroblast marker - FSP1. *J. Cell Biol.* 130 393–405. 10.1083/jcb.130.2.393 7615639 PMC2199940

[B79] Sundberg C. Nagy J. A. Brown L. F. Feng D. Eckelhoefer I. A. Manseau E. J. (2001). Glomeruloid microvascular proliferation follows adenoviral vascular permeability factor/vascular endothelial growth factor-164 gene delivery. *Am. J. Pathol.* 158 1145–1160. 10.1016/s0002-9440(10)64062-x 11238063 PMC1850349

[B80] Tal R. Shaish A. Rofe K. Feige E. Varda-Bloom N. Afek A. (2008). Endothelial-targeted gene transfer of hypoxia-inducible factor-1 alpha augments ischemic neovascularization following systemic administration. *Mol. Ther.* 16 1927–1936. 10.1038/mt.2008.191 28189008

[B81] Torchilin V. P. (2005). Recent advances with liposomes as pharmaceutical carriers. *Nat. Rev. Drug Discov.* 4 145–160. 10.1038/nrd1632 15688077

[B82] Tuder R. M. Chacon M. Alger L. Wang J. Taraseviciene-Stewart L. Kasahara Y. (2001). Expression of angiogenesis-related molecules in plexiform lesions in severe pulmonary hypertension: evidence for a process of disordered angiogenesis. *J. Pathol.* 195 367–374. 10.1002/path.953 11673836

[B83] Ueno M. Maeno T. Nomura M. Ikeda K. Aizawa T. Yamaguchi S. (2009). HIF-1a promotes pulmonary fibrosis through PAI-1 production. *Am. J. Respir. Crit. Care Med* 179:A3423 10.1164/ajrccm-conference.2009.179.1_meetingabstracts.a3423

[B84] Veith C. Schermuly R. T. Brandes R. P. Weissmann N. (2016). Molecular mechanisms of hypoxia-inducible factor-induced pulmonary arterial smooth muscle cell alterations in pulmonary hypertension. *J. Physiol. London* 594 1167–1177. 10.1113/jp270689 26228924 PMC4771790

[B85] Veith C. Zakrzewicz D. Dahal B. K. Balint Z. Murmann K. Wygrecka M. (2014). Hypoxia- or PDGF-BB-dependent paxillin tyrosine phosphorylation in pulmonary hypertension is reversed by HIF-1 alpha depletion or imatinib treatment. *Thromb. Haemost.* 112 1288–1303. 10.1160/th13-12-1031 25231004

[B86] Wang G. L. Jiang B. H. Rue E. A. Semenza G. L. (1995). Hypoxia-inducible factor-1 is a basic-helix-loop-helix-pas heterodimer regulated by cellular o-2 tension. *Proc. Natl. Acad. Sci. U.S.A.* 92 5510–5514. 10.1073/pnas.92.12.5510 7539918 PMC41725

[B87] Wang J. Fu X. Yang K. Jiang Q. Chen Y. Jia J. (2015). Hypoxia inducible factor-1-dependent up-regulation of BMP4 mediates hypoxia-induced increase of TRPC expression in PASMCs. *Cardiovasc. Res.* 107 108–118. 10.1093/cvr/cvv122 25824146 PMC4560045

[B88] Wang J. Weigand L. Lu W. J. Sylvester J. T. Semenza G. L. Shimoda L. A. (2006). Hypoxia inducible factor 1 mediates hypoxia-induced TRPC expression and elevated intracellular Ca2+ in pulmonary arterial smooth muscle cells. *Circ. Res.* 98 1528–1537. 10.1161/01.res.0000227551.68124.98 16709899

[B89] Wang L. Bell P. Morizono H. He Z. Pumbo E. Yu H. (2017). AAV gene therapy corrects OTC deficiency and prevents liver fibrosis in aged OTC-knock out heterozygous mice. *Mol. Genet. Metab.* 120 299–305. 10.1016/j.ymgme.2017.02.011 28283349 PMC5423267

[B90] Wang Y. Saad M. Pakunlu R. I. Khandare J. J. Garbuzenko O. B. Garbuzenko O. B. (2008). Nonviral nanoscale-based delivery of antisense oligonucleotides targeted to hypoxia-inducible factor 1 alpha enhances the efficacy of chemotherapy in drug-resistant tumor. *Clin. Cancer Res.* 14 3607–3616. 10.1158/1078-0432.ccr-07-2020 18519795

[B91] Wasko P. Luchowski R. Tutaj K. Grudzinski W. Adamkiewicz P. Gruszecki W. I. (2012). Toward understanding of toxic side effects of a polyene antibiotic amphotericin B: fluorescence spectroscopy reveals widespread formation of the specific supramolecular structures of the drug. *Mol. Pharm.* 9 1511–1520. 10.1021/mp300143n 22506900

[B92] Wenger R. H. (2002). Cellular adaptation to hypoxia: O-2-sensing protein hydroxylases, hypoxia-inducible transcription factors, and O-2-regulated gene expression. *FASEB J.* 16 1151–1162. 10.1096/fj.01-0944rev 12153983

[B93] Whitfield M. L. Finlay D. R. Murray J. I. Troyanskaya O. G. Chi J. T. Pergamenschikov A. (2003). Systemic and cell type-specific gene expression patterns in scleroderma skin. *Proc. Natl. Acad. Sci. U.S.A.* 100 12319–12324. 10.1073/pnas.1635114100 14530402 PMC218756

[B94] Wipff J. Dieude P. Avouac J. Tiev K. Hachulla E. Granel B. (2009). Association of hypoxia-inducible factor 1A (HIF1A) gene polymorphisms with systemic sclerosis in a French European Caucasian population. *Scand. J. Rheumatol.* 38 291–294. 10.1080/03009740802629432 19306159

[B95] Wojciak-Stothard B. Abdul-Salam V. B. Lao K. H. Tsang H. Irwin D. C. Lisk C. (2014). Aberrant chloride intracellular channel 4 expression contributes to endothelial dysfunction in pulmonary arterial hypertension. *Circulation* 129 1770–1780. 10.1161/circulationaha.113.006797 24503951 PMC4033409

[B96] Xu W. Erzurum S. C. (2011). Endothelial cell energy metabolism, proliferation, and apoptosis in pulmonary hypertension. *Compr. Physiol.* 1 357–372. 10.1002/cphy.c090005 23737177 PMC6467053

[B97] Xu W. Koeck T. Lara A. R. Neumann D. DiFilippo F. P. Koo M. (2007). Alterations of cellular bioenergetics in pulmonary artery endothelial cells. *Proc. Natl. Acad. Sci. U.S.A.* 104 1342–1347. 10.1073/pnas.0605080104 17227868 PMC1783136

[B98] Yu A. Y. Shimoda L. A. Iyer N. V. Huso D. L. Sun X. McWilliams R. (1999). Impaired physiological responses to chronic hypoxia in mice partially deficient for hypoxia-inducible factor 1 alpha. *J. Clin. Investig.* 103 691–696. 10.1172/jci5912 10074486 PMC408131

[B99] Yun X. Maman S. Jiang H. Shimoda L. (2015). HIF upregulates AQP1 expression in pulmonary arterial smooth muscle cells (PASMCs) in a calcium-dependent manner. *FASEB J.* 29(Suppl. 1031.8).

[B100] Zeisberg M. Kalluri R. (2004). The role of epithelial-to-mesenchymal transition in renal fibrosis. *J. Mol. Med.* 82 175–181. 10.1007/s00109-003-0517-9 14752606

[B101] Zeng Y. Liu H. Kang K. Wang Z. Hui G. Zhang X. (2015). Hypoxia inducible factor-1 mediates expression of miR-322: potential role in proliferation and migration of pulmonary arterial smooth muscle cells. *Sci. Rep.* 5:12098 10.1038/srep12098 PMC4499844 26166214

[B102] Zhang Q. Z. Wu Y. D. Ann D. K. Messadi D. V. Tuan T. L. Kelly A. P. (2003). Mechanisms of hypoxic regulation of plasminogen activator inhibitor-1 gene expression in keloid fibroblasts. *J. Investig. Dermatol.* 121 1005–1012. 10.1046/j.1523-1747.2003.12564.x 14708599

[B103] Zhang Q. Z. Wu Y. D. Chau C. H. Ann D. K. Bertolami C. N. Le A. D. (2004). Crosstalk of hypoxia-mediated signaling pathways in upregulating plasminogen activator inhibitor-1 expression in keloid fibroblasts. *J. Cell. Physiol.* 199 89–97. 10.1002/jcp.10452 14978738

[B104] Zhou G. Dada L. A. Wu M. Kelly A. Trejo H. Zhou Q. (2009). Hypoxia-induced alveolar epithelial-mesenchymal transition requires mitochondrial ROS and hypoxia-inducible factor 1. *Am. J. Physiol. Lung Cell. Mol. Physiol.* 297 L1120–L1130. 10.1152/ajplung.00007.2009 19801454 PMC2793183

